# Editorial: Non-coding RNA and renal disease

**DOI:** 10.3389/fmolb.2024.1404930

**Published:** 2024-05-10

**Authors:** Cristina Espinosa-Diez, Verónica Miguel, Nik Tsotakos

**Affiliations:** ^1^ Center for Molecular Medicine and Genetics, Wayne State University, Detroit, MI, United States; ^2^ Spanish National Center for Cardiovascular Research (CNIC), Madrid, Spain; ^3^ Department of Biological Sciences, School of Science Engineering and Technology, Penn State Harrisburg, Harrisburg, PA, United States

**Keywords:** microRNA, lncRNA, transcriptomics, acute renal injury, kidney renal clear cell carcinoma, diabetic kidney disease, nephrotoxicity, biomarker

Advancements in high-throughput sequencing have allowed the decoding of non-coding regions in the genome. They comprise diverse regulatory and functional units, among the most numerous loci encoding non-coding RNAs (ncRNAs).

Growing numbers of ncRNAs, such as miRNAs and long non-coding RNAs (lncRNAs), have been linked to human diseases, including those related with the kidney. However, their functionality remains controversial, and the number of experimentally characterized or disease-associated ncRNAs is still limited.

The Research Topic “Non-Coding RNA and Renal Disease” presents novel techniques and approaches, including ncRNA transcriptomic screening, dataset analysis for differentially expressed ncRNA identification, ncRNA interaction with RNA and proteins as well as its impact on the epigenetic landscape, to decipher the functional role of ncRNA regulators in a cell and stage-specific manner during kidney disease development.

This article Research Topic comprises four original research articles elucidating new molecular mechanisms driven by ncRNA dysregulation that underlie the pathology of representative kidney diseases, such as acute kidney injury (AKI), kidney renal clear cell carcinoma (KIRC), diabetic kidney disease (DKD), and nephrotoxicity induced by the pollutant bisphenol A. The model systems range from cellular platforms and transgenic mice, along with renal injury models and human biopsies from patients, facilitating the translation of research findings into the clinical practice as biomarkers or therapeutics.

The article by Qiu et al. identifies a novel adipokine termed C1q And TNF Related 1 (C1QTNF1), whose levels in tumor tissue from KIRC patients correlate with cancer progression and immune cell infiltration. Of note, increased expression of C1QTNF1 suggests a poor prognosis in these patients. The authors also demonstrate that the silencing of C1QTNF1 inhibits KIRC cell proliferation, cell migration, and cell invasion and propose the CYTOR and AC040970.1/hsa-miR-27b-3p axis as an upstream ncRNA-related pathway regulating C1QTNF1 in KIRC.

The study by Kato et al., explores the function of the long non-coding RNA megacluster (lncMGC) in DKD progression, particularly in response to elevated TGF-β in the glomeruli. Building on their previous discovery that inhibiting lncMGC mitigates DKD features, such as fibrosis and inflammation, the authors employed a multi-omics approach to identify protein partners of lncMGC that mediate DKD progression. They found that lncMGC is associated with various nucleosome remodeling factors, including SMARCA5 and SMARCC2. This interaction elucidates an epigenetic mechanism by which lncMGC influences gene expression, enhancing chromatin accessibility at sites of TGF-β-responsive genes implicated in DKD progression. Additionally, lncMGC is capable of autoregulating its promoter region. These insights underscore the pivotal role of lncRNAs in epigenetic regulation and their potential as therapeutic targets in DKD.

The article by Tao et al. provides insights into the miRNA-mRNA regulatory networks involved in ferroptosis and identifies a potential therapeutic target for AKI caused by renal ischemia-reperfusion. This study demonstrated that inhibition of miR-3587 can attenuate ferroptosis following renal ischemia-reperfusion injury by upregulating heme oxygenase-1 (HO-1), a crucial inhibitor of ferroptosis, thereby protecting renal tissues from injury. The inhibition of miR-3587 also resulted in increased GPX4 protein levels, enhanced cell viability, reduced malondialdehyde content, decreased Fe^2+^ levels, and restoration of normal mitochondrial membrane potential, indicating a protective effect against ferroptosis. Transmission electron microscopy analysis also showed improved mitochondrial integrity. The authors utilized bioinformatics analysis, *in vitro* models, and luciferase reporter gene assays to confirm the direct regulation of miR-3587 on HO-1 expression. These findings contribute to understanding the pathophysiological mechanisms of AKI and offer a potential treatment strategy for patients at risk of renal ischemia-reperfusion injury.

In the study by Wiszpolska et al., 433 genes and 95 long-noncoding RNAs showed differential expression in kidneys exposed to bisphenol A (BPA), a ubiquitous endocrine disruptor in plastic manufacturing, with harmful effects on health. The research aims to explore the molecular changes induced by oral BPA exposure in mouse kidneys through a comprehensive transcriptomic analysis. Using 12 female mice, divided equally between the control group and the BPA-exposed group, which received BPA in their drinking water for 3 months, the authors performed bulk RNA sequencing from kidney tissues, revealing differential gene expression in BPA-treated kidneys, indicating significant changes in processes like oxidative phosphorylation, mitochondrial function, and chemical carcinogenesis. Interestingly, 80 correlation pairs of differentially expressed coding and non-coding genes were identified, but none of these non-coding RNA genes were located within 10 kb of the differentially expressed coding genes. Despite the ongoing challenge of non-coding RNAs not being included in network analysis databases, the study concludes that BPA adversely affects kidney function by inducing mitochondrial dysfunction, which leads to oxidative stress and reactive oxygen species production.

In conclusion, this “Non-Coding RNA and Renal Disease” editorial Research Topic illuminates the profound and multifaceted roles that ncRNAs play in the pathology of various kidney diseases. Through an array of innovative research methodologies, this body of work expands our understanding of ncRNA mechanisms in disease processes and paves the way for novel diagnostic and therapeutic strategies. From the intricate regulatory networks involving miRNAs and long non-coding RNAs to the identification of potential biomarkers and therapeutic targets, each study contributes significantly to the knowledge of kidney disease at the molecular level. The insights from these investigations underscore the critical importance of ncRNAs in kidney health and disease, offering hope for developing more effective treatments for conditions such as acute renal injury, kidney renal clear cell carcinoma, diabetic kidney disease, and nephrotoxicity. As research in this field continues to evolve, the potential for ncRNAs as key players in the future of renal disease management and therapy becomes increasingly evident.

